# The Hospital. Nursing Section

**Published:** 1904-04-16

**Authors:** 


### The Hospital.

**Nursing Section.**

**Contributions for this Section of "The Hospital" should be addressed to the Editor, "The Hospital" Nursing Section, 28 & 29 Southampton Street, Strand, London, W.C.**

No. 916.—Vol. XXXVI. *SATURDAY, APRIL* 16, 1904.

### Notes on News from the Nursing World.

### ROYAL NATIONAL PENSION FUND FOR NURSES.

The annual general meeting of the members of the Royal National Pension Fund for Nurses will be held at 4 P.M. on Thursday, April 21st, at River Plate House, Finsbury Circus, E.C. It is the wish of the council that as many policy-holders as possible should attend. All that is necessary for those who accept the invitation is to write their names and policy numbers legibly on a card to be handed in on entering. The proceedings on the occasion are always full of interest, and it will be worth the while of some who have never been present to make an effort to put in an appearance next Thursday.

### QUEEN ALEXANDRA'S MILITARY NURSING SERVICE.

Miss Gertrude Mary Richards has been appointed matron in Queen Alexandra's Imperial Military Nursing Service. Miss C. H. Potts has been promoted from the rank of sister to that of matron. Miss C. P. Gash, and Miss E. M. Monck-Mason, sisters, and Miss E. S. Mason and Miss M. Walker, staff nurses, are confirmed in their appointments, their period of provisional service having expired. Miss C. C. Richards Moor has resigned her appointment as staff nurse.

### THE DUCHESS OF ALBANY AND NURSE POW.

The late Miss Jane Pow, who has just died at the age of 81, was not, of course, trained on modern lines, but the Duchess of Albany, who had special opportunities of judging, considered that she was a born nurse. It was in recognition of Miss Pow's untiring attention to the Duke of Albany during the illness which terminated in his death that Queen Victoria presented Miss Pow with a valuable gold Lorne brooch. Miss Pow, who was born at Penicuik and is laid to rest there, began nursing in the sixties, and thanks to the admirable manner in which she discharged her duties, her services were in great request for some years by distinguished persons at home and abroad. She only retired from work thirteen years ago.

### ENDOWMENT OF A DISTRICT BY MRS. WILLIAM RATHBONE.

It was announced at the annual meeting of the subscribers to the Liverpool Queen Victoria Nursing Association that Mrs. William Rathbone had generously endowed her district in memory of the late Mr. William Rathbone with a gift of £1,000, which had been invested. Henceforth this district will accordingly be known as the "William Rathbone Memorial District," and the hope was expressed at the meeting that others might see their way to a similar benefaction. The need of such benefactions is certainly very great, for the debit balance of the association at the end of 1903 was £1,274. This, the treasurer explained, was solely accounted for by the expansion of the work, no extraordinary expenses having been incurred. The figures show that the cases nursed were 7,309, an increase of 60, and the visits paid 176,279, an increase of 5,361. In addition to these the nurses made 55,673 dressings in attending the minor ailments of children in the elementary schools. Mr. John Henderson said that he should like to keep the association free from anything in the nature of parish relief, and the best way to avoid an appeal to the guardians for help is to induce a number of well-to-do Liverpudlians to emulate the admirable example of Mrs. Rathbone.

### A NURSE AND HER INSTRUCTIONS.

Nurses will be naturally much interested in the remarkable state of affairs now existing at Bolingbroke Hospital, Wandsworth Common, as revealed by the evidence of Miss Ormonde at an inquest on Monday in an account in another column. There are responsibilities which ought not, under any circumstances, to be imposed upon them. One of these is the burden of deciding whether a patient is better or worse, or ill enough to become an in-patient, which should not fall upon the nurse in attendance in the out-patient department. At the same time we do not see how Miss Ormonde could have refused to do as she was told by her official superiors. It is the primary duty of a nurse to obey instructions, and if the instructions are wrong the blame attaches to those who give them, not to the person who carries them out.

### THE CROYDON INFIRMARY CERTIFICATE.

Last week we suggested that as Croydon Workhouse Infirmary is a training school for nurses recognised by the Local Government Board, it is time that the supreme authority intervened and settled the question of the matron's signature to certificates once for all. The fact that a probationer on trial has left Croydon Infirmary because her father had ascertained that its certificate would not qualify her for admission to Queen Alexandra's Imperial Military Nursing Service, ought to have finally convinced the Croydon Guardians of the folly of not at once reinstating the matron's signature, but a ridiculous suggestion is now before them that the matron shall be authorised to sign a supplementary certificate, if she desires to do so. The adoption of this proposal would, instead of improving matters, make them worse. The only proper course is to follow the procedure which obtains at other large workhouse infirmaries, and until this has been done, it certainly cannot be wished that the Croydon Guardians should be able to command the services of the best class of nurses.

### AN AMERICAN DIFFICULTY.

There has been an interesting discussion at Pittsburg respecting the question "of caring for nurses suffering from contagious diseases." A proposal was made to build a special cottage hospital somewhere in the suburbs, but this was discarded as entirely impracticable, and it is probable that an apartment for the purpose will be secured in the new municipal hospital. Action in the matter has become necessary "because of the difficulty of obtaining a bed in the local hospital for a nurse contracting a contagious disease."

### PLYMOUTH GUARDIANS AND THEIR CHARGE NURSES.

A fortnight ago we referred to the fact that a committee of Plymouth Guardians had been directed to inquire into the reasons why two candidates had refused to take up the appointment of charge nurse at the workhouse infirmary. The committee met, but have been unable to discover any reason why the nurses refused the positions, but the Governor has stated that neither the hospital, nurse, nor anybody connected with the house had anything to do with the matter. The refusals, now increased to four, are all the more remarkable on that account. Meanwhile, the superintendent nurse has written to the Guardians that, "after such reports appearing in the nursing journals and various other papers, and having already written the Infirmary Committee and received no reply, she begs to say that if they wish her to remain in their service, she must ask that either a definite complaint be made and an impartial inquiry held, or that insinuations be avoided, as they are most objectionable to herself and very detrimental to the working of the infirmary." At the last meeting of the Guardians this question was discussed at some length, and Mr. J. S. Argall made a speech in which he affirmed that the nurses "have duties put upon them which one would not give to a domestic servant." Other reasons given are the entire lack of the comforts of home, and the difficulties which are experienced in obtaining a testimonial. If these things are so, it is highly probable that they came to the ears of newly-appointed charge nurses, who, accordingly, consider it undesirable to take the risk of going to Plymouth. The Hospital Committee, it may be hoped, will get at the truth, and report without fear or favour.

### THE CLASSES JUST ABOVE "THE VERY POOR."

A point which is often missed at meetings of nursing associations was emphasised at the annual meeting of the South London District Nursing Association. Mrs. Stanley Boyd, M.D., in urging the importance of the nurses attending the classes just above what is generally meant by "the very poor," described them as "the classes who can manage to give small donations to the Association, but who can no more afford to pay the ordinary fees for private nurses than the poorest of the patients nursed by the Association." This is quite true, and it is a conclusive argument in favour of seeking financial support for the work of district nursing, not by levying fees, however small, but by inviting people who, though not "the very poor," have not means to pay for a nurse, to contribute to the funds. We believe that if this policy were more steadily and energetically pursued, district associations would be less frequently reproached with neglecting cases, and would also find their income materially augmented.

### THIRTY-FIVE YEARS A NURSE.

The Brighton Guardians have expressed their regret that owing to the age of Miss Emma Brown it has become necessary for her to retire. Miss Brown has served the Board for thirty-five years, most of the time as charge nurse, and, it is stated, has discharged her duties during the whole period very satisfactorily. She abundantly merits the testimonial which the guardians have agreed to give her. Her fine record is all the more noteworthy and creditable because, as the Brighton Guardians now admit, the nursing staff has been much overworked, some of the nurses at the old infirmary having had to be on duty 79 hours a week. Perhaps the retirement of Miss Emma Brown may have brought home to the guardians the improbability of finding another woman prepared to work under the same conditions. Certainly, the event coincides with their determination to augment the staff at the old infirmary. The appointment of a trained nurse, for alternative day and night duty on the male side, and of one probationer is, at any rate, a step in the right direction.

### ALICE MEMORIAL HOSPITAL, HONGKONG.

At the annual meeting of the finance committee of the Alice Memorial and Nethersole Hospitals, Hongkong, held on February 19th, reference was made to the death of Mrs. H. D. Stevens, matron of the hospital since 1891. In the report adopted at the meeting Mrs. Stevens was described as "a faithful and efficient nurse," and satisfaction was expressed that the committee were able to command the services of Miss Langdon, who had come as a voluntary worker to help Mrs. Stevens.

### AN UNFORTUNATE COMPARISON.

The chairman of the meeting of subscribers to the Cardiff District Nursing Association, in stating that during the year 1903 the ten nurses had paid upwards of 54,000 visits, an average of 5,000 each, said that they only cost the Association 4½d. each, whereas "if 5,000 visits were paid by a medical man he would be remunerated to the extent of at least £1,000 a year." This is either a mischievous or a futile comparison—mischievous if it suggests that the nurse may take the place of a medical man, and futile otherwise. The Queen's nurses at Cardiff, we presume, work there as elsewhere under the directions of the medical men, and Dr. Garrett Horder testified that he had "himself received great and beneficial assistance from them." The function of the nurse is here accurately indicated by Dr. Horder; she is the assistant, not the substitute, of the doctor, and the cost of her attendances has nothing whatever to do with the amount of fees properly paid to medical men for their entirely different services.

### NURSES' LEAVE AT HOLBORN INFIRMARY.

At a meeting of the Holborn Guardians, Miss Baker opposed a recommendation to extend the leave of the nurses on the ground that a new set of regulations had only recently been drawn up, and now it was sought to alter them. But as the chairman states that these regulations have not yet been ratified by the Board, we do not see the force of Miss Baker's objection. It is proposed by the Infirmary Visiting Committee that instead of the usual half-day's leave twice a month, the nurses, if they desire, should be permitted to go off duty at 8 p.m. one day, and return at 11 p.m. on the following day. This would mean the extension of leave from two half-days a month to one day and a half. The consideration of the recommendation has been deferred, but we hope, for the sake of those nurses whose friends live at a distance and cannot be visited in half a day, that it will be agreed to.

### THE NORFOLK AND NORWICH STAFF OF TRAINED NURSES.

On Thursday last week, 30 out of the 50 private nurses belonging to the Norfolk and Norwich Staff of Trained Nurses, assembled at 50 Bethel Street Norwich, to congratulate their lady superintendent on the 20th anniversary of her connection with the institution. The absentees sent letters and united in the present given by the staff, which consisted of a Queen Anne teapot, an oak tray with a double silver rail, and two rose bowls. The lady superintendent of the Lowestoft Branch Home gave a silver sugar-tongs, the secretary and a friend a silver hot-water jug, and Miss Watson was the recipient of numerous gifts from other friends, and from the servants of the home. The committee presented a gold medal, bearing the inscription, "Presented to Miss Edith Watson in grateful appreciation of her 20 years' valuable services as Lady Superintendent," and on the obverse the monogram and motto of the staff, "With Goodwill doing Service." Miss Warnes, a member of the committee since 1866, claimed the right to pin on the medal, and in a few well-chosen words, referred to the excellent work done by Miss Watson, and the esteem in which she is held by the nurses and the committee. Under her care, the staff has risen from 20 to 50, and within the last two years a branch home has been opened in Lowestoft and a district home in Norwich. Two of the nurses have been awarded gold medals for 20 years' service, and a third is due in May.

### PRIVATE AND DISTRICT NURSING AT LEICESTER.

That the services of the nurses attached to the private branch of the Leicester Trained Nurses' Institution are thoroughly appreciated is shown by the fact that the income for 1903 was £1,847. But the report which was read at the annual meeting says that the lady superintendent had to refuse applications for as many as 126 cases because of the inadequacy of the staff. Why is the staff inadequate? Cannot trained nurses in sufficient abundance be obtained, or do the fees received fail to suffice to cover the expense of the branch? Clearly, the fees earned by the private nurses are not devoted to the purpose of keeping the district branch in funds, for in that branch there is a balance on the wrong side of no less than £161. This should not be the case in prosperous Leicester with its thousands of artisans earning good wages.

### DISTRICT NURSING AT CAMBRIDGE.

There are several gratifying facts to note in connection with the annual meeting of the Cambridge District Nursing Institution. The number of visits paid by the nurses during 1903 was 5,813, as against 4,488 in 1902; the receipts for the 12 months amounted to £1,253, and the balance in hand was £74. The Rev. Dr. Cunningham, vicar of Great St. Mary's, was able to give his personal testimony as to the extraordinary influence which the nurses exercised by their visits. It had been brought home to him in a case where Addenbrooke's Hospital was no longer able to take charge of a patient who was very seriously ill, because it was feared that he might affect other cases. He was, therefore, compelled to return to his home, and the district nurse had not only attended him, but had also prevented his children from manifesting their devotion to him by sitting in his room all night, and undertaking services which, though well intended, were all in the wrong direction. There are now six nurses, in addition to the lady superintendent, residing in the home at Cambridge; and so great is the confidence of the Board of Guardians in the nurses of the institution that they have given into its hands the whole of their outside nursing.

### PROGRESS AT HAMPTON.

The report of the committee of the Hampton Nursing Association for 1903 shows not only that admirable work has been done, but also that the position of the organisation is financially sound. During the year nearly 4,000 visits were paid, and 236 patients visited. We note the report from the inspector from headquarters that the nurses' work is "very satisfactory," but if, as the committee anticipate, the operations of the association are likely to increase, she will need a colleague. Happily, there is a balance in the bank of £100, and though this is £13 less than at the end of 1902, it indicates a condition of prosperity which augurs well for an extension of the work.

### MUSIC AND NURSING.

The treasurer of the Shoreditch and Bethnal Green Nursing Association has received from Mr. Edward Gates a cheque for £71 5s., which represents the proceeds of a charity given in Shoreditch Town Hall on behalf of the organisation. We are glad to hear that the promoters intend to make the event an annual fixture.

### SHORT ITEMS.

It has been decided by the committee of Lady Dudley's Fund for the maintenance of district nurses in the poorest parts of Ireland to send out a tenth nurse at an early date.—The result of the variety entertainment at Wokingham a few weeks ago [illegible] that the handsome sum of £60 10s. was realised a[illegible] has been handed to the Benefit Nursing Associatio[illegible]

### The Nursing Outlook.

"From magnanimity, all fear above;

From nobler recompense, above applause,

Which owes to man's short outlook all its charm."

### LIGHT TREATMENT.

The need for nurses to be well educated is demonstrated most fully by the perpetual increase in the number of forms of Light treatment, and the rapid discoveries that are being made along this line of cure. For how is a nurse to be the intelligent handmaid of the doctor in the Light department if she has no understanding of the physical properties of heat and light? It used to be urged with regard to the "lady" nurse fifty years ago that her accomplishments would all be beneficial in the hospital ward, that she could sing to the patients or play the piano at prayers, that she could paint bulrushes on the ward doors, and work table-cloths for the ward tables. It can equally be urged now that the scientific training of the High School girl all fits her to take her part should she later on desire to become a nurse. Indeed, no knowledge is ever lost, and it has been most truly said that if only we keep our tools ready God will find us work.

It was just ten years ago that Dr. Finsen's experiments with Light as a therapeutic agent began to be talked about in England; and it was in 1896 that Roentgen discovered a type of ray which is capable of passing through opaque substances, and called these the *x*-rays. Neither the Finsen rays nor the *x*-rays are visible to the eye, and their existence and powers can only be explained properly to those who have some knowledge of physics. Now every leading hospital has its Finsen or French lamp for lupus cases, and its *x*-ray apparatus, and nurses had just begun to be familiar with these new appliances and to comprehend a little of the science of the treatment, when the discovery of radium by Mme. Curie once more raised new questions and new hopes in the medical world. Luckily in London there have been several very excellent lectures on the various rays and radium, and most nurses have been able to attend and hear and see. The experiments are a wonderful help at these lectures, but unless they can be well seen it is very difficult to follow, and with the small quantities of radium at our disposal, it has been impossible to demonstrate satisfactorily before very large audiences.

Still, the London nurse has many advantages over the provincial nurse, who is usually unable to gain experience of Light treatment in the course of her ordinary training, and who, owing to insufficient acquaintance with the fundamental principles underlying the subject, is quite unable to follow intelligently the accounts given in the newspapers and scientific journals. We suggest that rather than starting with radium the probationers should begin with Macmillan's shilling science primers and the apparatus named in them; a small physics laboratory can thus be fitted up for about five or ten pounds. Then surely the medical staff have, of necessity, studied the subject, and one of them could arrange three simple lectures on the work of Finsen, Roentgen, and the Curies? It even seems to us that it would be worth while for the sake of the experiments to secure the services of a really good lecturer, and to so arrange that it should be public, but that the nurses should have the benefit of good seats.

It may be urged that already from the medical point of view radium has proved a failure, and that its properties and uses are not necessary knowledge for a nurse. Probably in its exactness that is true, but it must be remembered that Light and Heat baths and institutes are springing up here and on the continent and in the colonies at a great rate, and that many of these institutions "train" their own nurses for three or six months, and that it is deplorable to hear some of the quackery that these women talk. It certainly affects the nursing profession adversely if those members of it, who are supposed to be trained in the newest treatments, betray an ignorance of the elements of science which would disgrace a Board School girl. Surely a knowledge of the different forms of Light treatment should be something superadded to the training of the probationer, and should be thorough and on a firm basis. If trouble is not taken to give the real nurse a knowledge of these subjects, we shall be deluged with sham nurses. And the results may be very deplorable, for we cannot help remembering how, when massage first became fashionable, and the hospitals were not quick enough in training true nurses to the work, what awful scandals resulted.

The nurse at a private case is liable nowadays to have some extraordinary "electrothermogen" sent to her with orders to give a Light or Heat bath, or to be asked to try the Light treatment on some growth or sore, on such days as the sun makes this possible without a lamp. Directions from the doctor are of course always given, but if there be no knowledge of the scientific basis of the treatment, and no training in its application, mistakes are almost certain to result. Then suppose one of the three or six months' special nurses is sent along, and the case demands not only the Light treatment but also nursing care generally, mistakes will again result. It should be enough to give a word of warning to awake hospital authorities to their responsibilities in this matter. And to nurses who value their three years' training and are proud of their profession we would say if you have not knowledge of Light treatment, ask your training school to supply you with it.

### The Modern Nursing of Consumption.

By Dr. Jane H. Walker, Physician to the New Hospital for Women, London, and Medical Superintendant of the East Anglian Sanatorium, Nayland, Suffolk.

### LECTURE III.

### Subacute Phthisis.

Under this category we have, as said above, those lingering cases which are most commonly met with. Such cases may last for years, getting, perhaps, at times worse, at times better, ultimately often rewarding our efforts by being completely ameliorated.

It is in the care of these cases that the real worth of a nurse comes out. It is comparatively easy for any nurse to look after an acute case of tuberculosis; if she be not attentive, resourceful, and intelligent in the case of a severe and rapid illness, she is not worthy of the name of a nurse.

It is quite another thing to nurse a patient with a lingering, and perhaps the nurse may think, a rather dull complaint, which lasts for months with no very exciting interludes of complications, or agitating times of retrogression. It is in these cases that tact, cheerfulness, and firmness are required. Some nurses will get bored with the sameness of the patients' condition, and if this is the case the patient will probably get equally bored with the nurse. Some nurses are devoted to their patients, and are never happy unless they are doing everything for them, and tend to coddle them too much, which is a mistake, for the very length of the illness predisposes the patients to develop readily into helpless and absolutely self-centred invalids.

Under the old *régime* a phthisis patient was an interesting invalid, kept in a stuffy room, fed up every two hours or so on invalid diet, taken out perhaps occasionally in a bath-chair, the nurse walking beside with a protecting hand on the hood. She could fuss over the patient all day, and had plenty of routine nursing duties with which to occupy herself. Moreover the patient was practically doomed from the first, and the nurse's duties became daily more arduous, and all the qualities required for a "good case" were quickly brought out.

Now these methods are changed, and unless the patient be very ill, most of the nurse's time really goes in carrying out a number of small, rather trifling details, which, unless she be naturally conscientious, and kept up to her work by a sense of the duty of doing thoroughly what she has undertaken, will become unutterably boring.

The three salient points of the present-day treatment of Consumption are:—Open air, rest, feeding.

*Open air* alone is not the most important part of the treatment; rest and feeding are of equal importance, indeed, rest of both body and mind may really be considered the greatest of the three.

The open air saves one source of trouble: instead of having a sick room with its half-drawn blinds, window open at the top, or fixed with some so-called hygienic arrangement of a board below, and a screen in front of the door, the windows are simply left open day and night, or better still, taken out altogether, and if the room is not large, the door should be left open too. Kind friends and anxious relations may feel horrified, and if they rather shun the sick room, so much the better for the patient's chance of complete rest and recovery.

This almost superabundance of fresh air may prove rather trying for the nurse, and in the winter might deter many nurses from undertaking the care of consumptive patients. Any nurse who has been unaccustomed to really fresh airy rooms will no doubt suffer for a day or two, but she may comfort herself by the reflection that she will very soon get used to it, and become, in her turn, a terror to her 'fresh-air-but-no-draught" friends; for she will find it impossible to sit in any room without the windows widely open.

Of course the nurse may wrap up under her clothes, but she must remember never to do her work huddled up in a shawl, and must never shiver, or look miserable, at least in the patient's presence. So far as the patient goes, the advantages of carrying out the open-air system are so apparent after the first few days that no difficulty is likely to be experienced with him.

*Food.*—The feeding of phthisis patients is almost always a difficulty, even in sanatoria, and still more in private nursing it will, at first at any rate, require all a nurse's firmness, tact, and encouragement, to make a patient take the prescribed amount of food. We shall return to this later on; here we will only say that three good plain meals a day are far better than small invalid meals given oftener. Everything provided at each meal must be finished, and no snacks allowed in between whiles.

*Rest.*—The amount of rest required by a patient will depend upon the pulse and temperature as a rule. For the first fortnight or so patients at a sanatorium are generally kept in bed entirely, no matter how slight their case. Of course, if not very ill they may be allowed upon a couch while the bed is being made, and in slight cases may take baths in the bath-room. An hour's absolute rest is insisted on in most sanatoria before dinner and supper.

In a sanatorium the question of visitors is of course settled by the doctor; in private nursing the nurse should also allow no visitors except by special permission. Much firmness will at times be required to keep well-intentioned but agitating friends from coming in to what is called "cheer" the patient. It is often found that visitors whom one would not judge to be specially exhilarating companions will manage to excite a patient, and lead to a rise of temperature and quick pulse. Some patients, whose cases are not particularly bad, will always be upset by much talking and the excitements of visitors. There is besides another danger in allowing many visitors either in a sanatorium or a private house, which is the chance of a visitor having a cold or influenza coming, or come, on. There is, we consider, no complication more to be feared for consumptives than influenza, and even a common cold will often undo the work of weeks. The nurse must therefore assure herself that a visitor has neither of these fell complaints before admiting him.

*Clothing.*—The majority of patients, especially male patients, err on the side of wrapping themselves up too much. The mere idea of a cough seems to lead to an immediate multiplication of woollen and flannel garments, chest protectors and mufflers. The consequence is that the skin is kept in a perpetually moist condition, with the pores constantly open, and so tends to become unduly sensitive to all changes of temperature. Warmth is certainly required in the winter in this changeable climate, but all unnecessary wraps should be avoided; it is not necessary for men patients to wear thick sweaters in addition to ordinary sleeping suits, nor for women to have more than thin flannel or silk nightgowns. When sitting up in bed a flannel or silk, or in the coldest weather a padded, dressing-jacket may be worn. The nurse must set an example of fortitude by not wrapping herself up, externally at least, and she will find that she will feel the cold less and be better in health if she restricts herself to one warm garment next the skin, addition, of course, to ordinary linen under-clothes, with, in winter, a cellular or woven bodice.

In spite, or perhaps we should rather say because, of the open air, colds are unknown things in sanatoria, and no nurses need fear this plague of our salubrious climate. Cold weather need not be synonomous with discomfort; patients are allowed hot-water bottles *ad lib.* when they are resting or lying in the shelters, and warm but light blankets need never be stinted on the beds. It will be a surprising thing to most nurses to find how little the patients feel the cold after the first day or two. The need for, and the benefit of, fresh air are so rapidly felt that patients may often be found who are not contented with having one side of their rooms entirely window, with large ventilators over the doors, but will keep the doors wide open too. We have heard a patient, and this in the depth of winter, say that she wished the outer wall of the room could be taken out.

Of the special details of nursing, both in sanatoria and in private, we shall deal in our next lectures.

### Popularising Nursing in the Army.

### THE NEW REGULATIONS.

New regulations for admission to Queen Alexandra's Imperial Military Nursing Service have just been issued, and we are able to give authoritative official details of the changes in the conditions, from which it will be seen that the Nursing Board now offers advantages to nurses far in excess of any which have been offered before. The Queen, as President of the Nursing Board, takes the keenest interest in the Service, and has given the new organisation the most valuable advice and assistance.

### Increased Salaries.

First and foremost, perhaps, is the question of pay, a subject to which we alluded a few weeks back. The Staff Nurse now enters the Service at an initial salary of £40 per annum, rising by yearly increments of £2 10s. to £45. When she becomes Sister her salary starts at once at £50, and by yearly additions of £5 at length reaches £65 a year. The next step upwards is Matron, with an initial salary of £75, mounting by yearly sums of £10 to £150, with the addition of charge pay of a sum not exceeding £30 per annum, according to the magnitude of her charge. Above this there are only two steps—Principal Matron, who receives £175 per annum to start with, which is increased each year by £10 till it attains £205, and Matron-in-chief who begins at £300 a year, and ends, after five years' service, at £350.

### Board and Lodging.

In addition to these sums, Matrons, Sisters, and Staff nurses are supplied with public quarters—or with lodgings and servants at the public expense—and with fuel and light. With regard to allowances in lieu of board and washing, 15s. a week at a home station, and 21s. a week at a station abroad, are granted to each member of the Service. All the members of the Military Nursing Service receive an annual uniform allowance of £9 abroad and £8 at home.

### Promotion.

An important point is that promotion is both certain and rapid. Sisters are promoted from Staff nurses, Matrons from Sisters, and the posts of Principal Matron and Matron-in-chief are also filled from the ranks below by special selection. The proportion of Sisters and Matrons is particularly large. In an ordinary hospital there are usually three or more Staff nurses to each Sister, so that the chance of a Staff nurse becoming a Sister is only one to three; in the military service there are only 208 Staff nurses to 102 Sisters (exclusive of South Africa), so that the chances of promotion are exactly doubled. The number of Matrons with respect to the entire staff is also a high one, for whilst the Staff nurses and Sisters together number 310, there are 29 Matrons and two principal Matrons, as well as the Matron-in-chief.

### Foreign Service.

All members of the Service proceed in due course abroad for foreign service, and thus have an opportunity of seeing new countries, mixing with differen races, and experiencing fresh conditions of life under the most favourable circumstances. A nurse who remains some time in the Military Service may in time become a much-travelled woman, for not only may she be sent to South Africa, Egypt, Malta, Gibraltar, Hong Kong, Bermuda, or Jamaica, but to Canada, Barbadoes, St. Lucia, Ceylon, and Singapore. At most of these stations Colonial allowance varying from £36 to £54 per annum is received in addition to ordinary pay. In time of war, when so many nurses are anxious to go on active service, so to assist to the utmost of their power the soldiers who are fighting in defence of their country, the members of the regular Service have the prior claim and are the first to proceed to the seat of war. In the event of hostilities the Sisters of the Service will be temporarily appointed matrons, and thus have an opportunity of fitting themselves for their matronly duties when their permanent promotion comes. There is a special allowance given when proceeding on active service, and also in addition a free kit as supplied to officers.

### Enlarging the Experience.

Formerly the military nurses had but few opportunities of gaining up-to-date experience after once they had entered the Service, but together with many other improvements this has been altered. The larger military hospitals in time of peace have now good facilities for professional work, and afford unique opportunities for nurses studying tropical diseases, and so fitting themselves for service abroad. They also give many opportunities for assisting at important operations, as well as subsequently nursing the patients. After a certain time in the Service, when it is deemed by the Nursing Board that an opportunity of studying the latest methods of nursing and of witnessing new and interesting operations would be beneficial to a Sister or a Matron, it is proposed that military nurses should for a time visit some of the largest civil hospitals, so that they may note the advances made in science and surgery, and the improvements in the nursing of certain diseases. This experiment has lately been put into practice, and has been attended with much success, the members of the Service being most kindly received and assisted in every way by the medical and nursing staff of the selected hospitals. They were allowed special leave for this purpose on full-pay and allowances.

### Other Advantages.

There are other advantages which nurses should consider. The work, although it is on occasions hard and trying, and has to be carried on in all climates, at other times is light and affords a great deal of delightful experience and variety. Soldiers are excellent well-disciplined patients, always grateful to the Sisters who look after them, and most willing to assist them in every way. The orderlies, who work under the Sisters and Staff Nurses, are as a rule willing and anxious to learn their work, and will it is hoped

### POPULARISING NURSING IN THE ARMY—*Continued.*

under the new rules make excellent nurses. They are now, as we stated some time ago, to be trained for three years, and at the expiration of this period receive a certificate if they have passed their examinations and are well recommended by their Medical Officers and Matrons.

### Pensions and Holidays.

The Pension, which can be claimed by voluntarily retiring at 50 years of age, or by compulsory retirement at 55, is calculated on the rate of pay at the time of retirement, and after 10 years' service would be 30 per cent. of such pay, with an additional 2 per cent. for each year of service in excess of 10, and a maximum of 70 per cent. of such pay. In any case of special devotion to duty, where a higher pension has not been obtained, a special pension not exceeding £50 a year may be granted. If disabled in the Service, however short the period of nursing may have been, some gratuity is awarded, the amount to be determined by the Secretary of State. When injury or sickness overtakes a member, certified by the regulated medical authority to have been caused by the Service, full pay may be issued for a period of 12 months and half pay for another period of six months. In exceptional cases half pay is extended for a further six months. When the injury or sickness is not caused by the Service, full pay may be granted for three months, and after 20 years' service two-thirds pay; or with less than 20 years' service half pay for a further period of three months. Ordinary leave of absence is granted in each financial year as follows:—Matron-in-chief, Principal Matron and Matron, each six weeks; Sister, five weeks; Staff nurse, four weeks. Pay may be granted for accumulated leave of absence during service at a station abroad. The Sisters and Staff nurses have three hours off duty every day, and a half-day once a week, and also from Saturday at 2 P.M. until Sunday 10 P.M. once a month.

### A Military Nursing Home.

A large Nursing Home will be opened in London when the new hospital attached to the Medical School at Milbank is completed, containing a home for special Sisters and Nurses, who will be sent from there to different stations when there are serious cases requiring nursing in the smaller hospitals where there is no regular staff. There will be a Nursing Staff attached to this hospital; and also a Home Hospital for the Sisters themselves who may be invalided home from abroad, and otherwise require care and nursing.

### Appointment of Candidates.

A candidate for the position of Staff nurse or Sister must be between 25 and 35 years of age, and possess a certificate of not less than three years' training and service in medical and surgical nursing in a civil hospital recognised by the Advisory Board. She must be of British parentage, and must satisfy the Board that as regards education, character, and social status she is a fit person to enter the Imperial Nursing Service. The appointment of a Staff nurse, during her first first six months, is of a provisional character. Sisters also, when appointed, will be at first appointed for six months only; later, a month's notice is all that is required to terminate an engagement. Vacancies in the Service in the grade of Staff nurse will be filled during the present month and throughout May, and the Matron-in-chief will be at 68 Victoria Street, London, S.W., every Tuesday and Thursday till the beginning of June, between the hours of 10 A.M. and 1 P.M., in order to see nurses who would like to know fuller details of the conditions of service.

### Operation for Stone in the Bladder.

### EXAMINATION QUESTIONS FOR NURSES.

The question was as follows:—In nursing a patient who had been operated on for stone in the bladder by the suprapubic method, how would you arrange his bed and the dressings to ensure the greatest possible degree of comfort—suppose the case to be in a household where neither money nor appliances are plentiful?

### First Prize.

A case of supra-pubic cystotomy in a private house may be nursed with as great a degree of comfort to the patient as in any nursing institution, and with a minimum cost for dressings and appliances. A single bedstead is advisable, with a firm mattress. The mattress should be protected with a mackintosh sheet. (This will be the most expensive item, but is absolutely necessary for the patient's comfort.) A sheet is placed over this and firmly tucked in all round. Then a small piece of mackintosh, with draw-sheet over it, is placed under the patient's hips. The upper bed-clothes should be warm but light, with a sheet next to patient. The head should be made comfortable, whilst a pillow under the knees tends to prevent patient from being restless. A cradle, to remove pressure of bed-clothes, is essential, and an excellent one can be procured for a few pence. An ordinary long broom-handle and two flat window-screen sticks are all that are required. The cradle is made by cutting the broom-handle into four pieces of equal length. Then nail each end of screen stick on to each piece, thus forming a cross-bar resting on two upright posts. Join the other three pieces in same way, lay the cross-bars one over the other, and nail loosely together in centre. This cradle can be adjusted to any length or width (an advantage over the ordinary ones). For the first twenty-four hours after operation the wound will require redressing about every four hours, after which time there is no necessity to use bandages or extensive dressings. A small piece of antiseptic gauze may be placed on the wound, covered with gutta-percha tissue and absorbent wool on top. Strapping may be used to secure the dressing in place. Two narrow strips of absorbent wool should be cut nine inches long. One end should be fluffed out and placed against either side of wound, the other ends being placed in two small enamelled basins, one on each side of the patient. In this way the wool strips act as a drain, collecting the urine as it escapes from the wound and conducting it into the basins. Thus the amount of urine passed can be accurately ascertained, and the patient's skin kept in a more comfortable condition than when covered with a sodden dressing. The skin round the abdomen and groins should be washed frequently with warm water and soap, thoroughly dried and rubbed with lanoline or zinc ointment. The dressing on the wound itself need only be changed once or twice a day. When such a case is nursed in this way there need be no fear of chafing or bedsores, as with a little care the bed can be kept spotlessly clean and dry. The patient may lie on either side, when only one basin would be necessary. A pickle-bottle or small basin would receive the urine when passed naturally.—Kerrie.

### Second Prize.

I would arrange the bed, cover it all with mackintosh or American cloth, then a smaller mackintosh for under the draw-sheet, blankets to divide in the middle, a light single one to cover all; a cradle—or stool if nothing else can be got—then over that sheet, blankets, and quilt as required. See that patient is comfortable, not too warm, try to keep sick-room at even temperature to guard against chills. Patient should have a chest protector on made of gamgee, or cotton wool covered with gauze does very well, and a flannel jacket turned back to front, which is easier to change and less trouble to the patient. Dressing: Sterilised gauze several layers, gamgee or cotton wool sterilised right across the abdomen, with a piece of cotton wool at each side to absorb the urine. Wood wool is more absorbent, but more expensive. To sterilise dressing cut up gauze and gamgee required length, wrap in a clean towel, and put in the kitchen oven on a dish. Patient's skin will require to be watched; wash with soap and water frequently and wipe with a soft towel. Methylated spirits, vaseline, or powder may be applied. Powder, starch, zinc, and boracic, equal parts mixed.—Estelle.

### The Prize Winners.

The answers are not very good this month. The question was simple, but it touched upon a matter of nursing that requires much care and practical thoughtfulness on the part of a nurse. Not one of the papers is first class, "Kerrie" and "Estelle's" represent the best, but they leave much to be desired. They have been successful because they both mention the necessity of two mackintoshes, one that shall cover the whole bed and another that shall be removable with the draw-sheet. This is an important point because if for any reason (such as the undesirability of waking the patient from a refreshing sleep) the dressings and draw-sheet should become fully saturated the region of the bed beyond the small mackintosh and draw-sheet would become wet, uncleanly, and unwholesome. Their papers, however, show errors. It is a grave one on "Kerrie's" part to speak of fastening on a supra-pubic dressing with strapping!

### Honourable Mention.

This is gained by "Aubrey," "Daisy," and "Nancy." "Aubrey's" paper would have gained the first prize but by an oversight he omitted to make any mention of mackintosh. "Daisy's" paper is good, but so carelessly and incorrectly written as to be impossible to print.

### Question for April.

State what curative treatment you would pursue in cases of slight and also of serious bed-sores.

N.B.—This question is the sequel to one put on October 3rd, 1903, and is given that it may coincide with that given to our nurses abroad for this month.

### Rules.

The competition is open to all. Answers must not exceed 500 words, and must be written on one side of the paper only. The pseudonym, as well as the proper name and address, must be written on the *same* paper, and *not on a separate sheet.* Papers may be sent in for fifteen days only from the day of the publication of the question. All illustrations strictly prohibited. Failure to comply with these rules will disqualify the candidate for competition. Prizes will be awarded for the two best answers. Papers to be sent to "The Editor," with "Examination" written on the left-hand corner of the envelope.

N.B.—The decision of the examiner is final, and no correspondence on the subject can be entertained.

In addition to two prizes honourable mention cards will be awarded to those who have sent in exceptionally good papers.

### To Nurses.

We invite contributions from any of our readers, and shall be glad to pay for "Notes on News from the Nursing World," or for articles describing nursing experiences, or dealing with any nursing question from an original point of view. The minimum payment for contributions is 5s., but we welcome interesting contributions of a column, or a page, in length. It may be added that notices of appointments, entertainments, presentations, and deaths are not paid for, but that we are always glad to receive them. All rejected manuscripts are returned in due course, and all payments for manuscripts used are made as early as possible after the beginning of each quarter.

### Appointments.

[No charge is made for announcements under this head, and we are always glad to receive, and publish, appointments. The information, to insure accuracy, should be sent from the nurses themselves, and we cannot undertake to correct official announcements which may happen to be inaccurate. It is essential that in all cases the school of training should be given.]

Central London Ophthalmic Hospital, Gray's Inn Road.—Miss L. A. Baskett, has been appointed matron. She was trained at King's College Hospital, and has since held the position of night superintendent at the Royal Hospital for Diseases of the Chest, City Road; home sister at the Royal Infirmary, Bristol; and sister at the Forester Hospital, Much Wenlock, Salop.

Inverness District Asylum.—Miss Adele Schneider has been appointed sister-in-charge of the male infirmary block. She was trained for three and a half years at the Royal Asylum, Glasgow, and also at the Western Infirmary, Glasgow, for four years. She holds the Medico-Psychological certificate.

Kingswood District Nursing Association, Bristol.—Miss Marlow has been appointed matron. She was trained at the Royal Hants County Hospital, Winchester. She has also been for four years at the Fountain Fever Hospital, Tooting, S.W.; one year at the Victoria Home, Broadstairs; and sister of the women's medical ward and night superintendent at the Royal Hants County Hospital, Winchester.

Maidenhead Union Infirmary.—Miss E. Constance Page has been appointed superintendent nurse. She was trained at the Greenwich Infirmary, has done private nursing, and been night superintendent at Her Majesty's Hospital, Stepney. She holds the L.O.S. certificate.

Middlesbrough Sanatorium.—Miss M. Porritt has been appointed charge nurse. She was trained at the Knightswood Hospital, Glasgow, and has since held the post of staff nurse at the Isolation Hospital, Scarborough, and Saltwell Hall Hospital, Gateshead.

North Devon Infirmary, Barnstaple.—Miss Lillie has been appointed matron. She was trained at the Victoria Hospital for Children, Chelsea, and at the Great Northern Central Hospital, Holloway, where she afterwards held the post of theatre sister. She has been night sister, ward sister, and home sister at the Hospital for Children, Shadwell. She holds the L.O.S. certificate.

Queen Alexander's Military Nursing Service for India.—Miss Ethel Kelso has been appointed nursing sister. She was trained at Charing Cross Hospital, where she has since been ward sister.

Western Fever Hospital, Fulham.—Miss Lucy Smith has been appointed charge nurse. She was trained at the Greenwich Infirmary, where she has on several occasions done head nurse's duties. She also holds the L.O.S. certificate.

### Where to Go.

Dowdeswell Galleries, 160 New Bond Street.—Mons. Lucien Monod is showing some beautiful crayon drawings, mainly studies of figures, at this gallery. This special style of drawing and portraiture was very much in vogue some 40 or 50 years ago, and the graceful method is again finding favour. Mons. Lucien Monod is a master in crayons, and his beautiful drawings are well worth a visit.

### Everybody's Opinion.

[Correspondence on all subjects is invited, but we cannot in any way be responsible for the opinions expressed by our correspondents. No communication can be entertained if the name and address of the correspondent are not given as a guarantee of good faith, but not necessarily for publication. All correspondents should write on one side of the paper only.]

### PORTRAITS AS PRESENTATIONS.

"A Reflective Pro" writes: The wisdom of the suggestion made in your columns that miniatures should be given as presentations is forcibly brought to my mind every day as I pass a large, and I should think expensive, photograph of a former matron of my hospital. At first, I daresay the photograph was very effective; but now, alas! owing to the action of the sun, it is so faded that it is almost a caricature of the original, who was a handsome woman. If, instead, her face had been reproduced in a miniature, it would have been as good to-day, or even better, than it was twenty years ago.

### STATE REGISTRATION.

Miss Sophie Rothwell, matron of Rochdale Infirmary, writes: I shall be much obliged if you will kindly add my name to the manifesto against the State registration of nurses. I am sure it would do no good and might do much harm.

Miss Florence K. Monkhouse, matron, Borough Fever Hospital, Ipswich, writes: If not too late I should like my name to be added to the manifesto against State registration of trained nurses. Like another correspondent, I feel sure that State registration will do more harm than good. We need protection against the so-called qualified nurse, but I am afraid that State registration will not give it us.

### PROTECTING MATTRESSES.

"J. H." writes: I have found mackintoshes and sheets slip under restless patients on spring beds, and so cause risk of accident to the mattress. I have been successful in keeping the mattress better covered with the mackintosh by sewing a deep piece of calico on the edge of the mackintosh, and tucking it well under. This only applies to the difficulties of spring beds, the old lathed ones were firmer. In the case of children's beds I have sewed the mackintosh all round the mattress, leaving only the ends open, and used the same mattresses for years.

### PRIVATE NURSING IN MADRAS.

Miss F. Dent, writes from the Nursing Home, Mackay's Gardens, Madras, under date of March 21st: My attention has been called to a slight inaccuracy in the paragraph under the above heading which you were good enough to insert *re* my nursing home in your issue of August 16th, 1903. What Mrs. Broadfoot stated in her letter to you dated December 9th, 1903, to the effect that she founded the nursing home in Mackay's Gardens in 1899 is quite correct The words "carry on" should be substituted for the word "found" in the last paragraph of the note which appeared in your columns. I have no wish to deprive Mrs. Broadfoot (*née* Brooke)—a lady nurse formerly of the General Hospital, Madras—of the credit of founding the home, nor of having nursed 173 patients therein during the four years it was under her management.

### FILLING A WATER-BED.

"Birse" writes: In answer to "M. G." on the above questions, as asked in The Hospital of date April 9th: This is how I saw it done for many years at a county asylum. The water-bed was placed on a mattress on the floor—not on the bed—so that the patient should not fall out, and when necessary another mattress alongside, so that if they did fall no harm could come to them, and filled with tepid water before the patient was laid on it. As I have been connected with very large asylums I have seen water-beds much in use, but I never once saw the patient on the bed first and then filled the bed with water.

"A Crumpsall Nurse" writes: In reply to "M. G.'s" question *re* the correct way of filling a water-bed, I should like to give my opinion in answer to her invitation, if it is of intesest to any of your readers. I have nursed many cases which required water-beds of various sizes, and have found that to slip one under a patient is the most easy way. The opening where one pours in the water is usually at one of the corners, and I place the water-bed so that the opening is at the foot of the bedstead, or furthest away from the seat of injury. I do not see how, being so placed, it can in any way disturb the patient while being filled, as only the extreme corner need be raised to allow the water to run in. There is no necessity for water to run down under the sheet if it is carefully poured in, or a towel held round the funnel during the process. "M. G." does not say anything about the temperature of the water. I was taught that it should be about 98·4°. Having filled the water-bed under the patient there is no necessity to move him again.

"Private Nurse" writes: I have filled many water-beds during my training at a large infirmary, where for the credit of the much-abused infirmary nurse, let me say, we never had such a thing as a bed sore, except those admitted ready made. We always filled the water-bed on an adjoining bedstead and lifted the patient carefully on to it. This plan always gave satisfaction. Some few months ago, however, at a private case where there seemed a great deal of difficulty in getting a second bedstead, the idea struck me (I thought it was my own patent) of slipping the water-bed under and filling it with the patient lying on it. But it did not work. I could not calculate whether it was full enough or too full, all the water being pressed to the edges of the water-bed by the 15-stone weight of my patient in the centre. So I was obliged to give the plan up, and insist on another bedstead being procured. It strikes me that perhaps the principle may be all right in theory, but it certainly does not work. I would much like to hear a patient's view on the subject, for when all is said and done he would be the best authority.

### TRAVEL NOTES AND QUERIES.

By Our Travel Correspondent.

Various Questions (Royal Oak).—1. It is not at all too hot in either Normandy or Brittany. 2. Brittany is not exactly bracing, but I think you would like the South. I should recommend the neighbourhood of Quimper, from which you can visit the magnificent coast scenery round the Pointe de Raz, and the villages there are very quaint, and you would see some of the "Pardons." Normandy is not so picturesque except as to its architecture, and would not, I think, suit you so well. Moreover, it is more expensive. If you take my advice you will not attempt to see the Channel Islands as well as France. It will cost a great deal, and they seem poor after Brittany. 3. You would go to South Brittany, Quimper, etc., *viâ* St. Malo. Fare, second return from London to St. Malo, £2 1s. Journey on according to route but at small cost. If you wish to combine the Channel Islands, by mentioning it at the time, I believe you can arrange to break the journey at Guernsey or Jersey on your return if you go by the London and South Western Railway Company. 4. When you have decided where you are going, I will tell you of hotels. Very few pensions exist in that part of the world, and none except in the well-frequented tourist centres, where you do not wish to be. You may reckon your hotel and minor expenses, exclusive of the journey, at from £8 to £10 each for the month. August is the most expensive travelling month. The cost of journey will vary; you must tell me where you decide to go first. 6. The cheapest route to Lausanne is *viâ* Dieppe, Paris, and Pontarlier—second return, £4 13s. 4d. Switzerland is cheap to live in, but the journey comes heavy. Still a month at 4 francs per day is only £4 10[illegible] each for a month; and reckoning £2 more, or even £3, for tips and excursions, it would but come to £12 3s. 4d. each. 7. Mid-Wales is not cheap. I doubt if it would not come to more to spend [illegible] month there than to go to Lausanne. I know of nothing really cheap there. When you have settled on your spot let me hear again.

### Echoes from the Outside World.

### The King and Queen in Denmark.

THE 86th birthday of King Christian was celebrated on Friday amid great popular enthusiasm. About noon his Majesty, accompanied by Queen Alexandra, appeared on the balcony of the Palace, and was loudly cheered by the large crowd which filled the Amalienborg Square. King Edward and Queen Alexandra afterwards appeared together, and they also received a cordial welcome. In the evening they were both present at the dinner party given by the Crown Prince Frederick in honour of his father's birthday. On Saturday the King and Queen and several of the Princes and Princesses visited the Light-cure Institute of Professor Finsen. The visit lasted more than an hour, and both the clinic and the laboratory were inspected. Queen Alexandra was presented with a bouquet by some British patients. Professor Finsen being too ill to receive the Royal guests at the Institute, they visited him at his own home, and complimented him on his great services to science. On Monday evening the King and Queen were present at a ball given by Prince and Princess Christian of Denmark.

### Agreement between England and France.

LAST week a new agreement between England and France was signed in London by Lord Lansdowne and M. Cambon, the French Ambassador. The agreement consists of three documents. One is a convention concerning Newfoundland and West Africa. In this case France abandons her exclusive right of fishing on the west shore, which has caused so much friction in the past. England concedes indemnities to the French fishing companies, a port on the river Gambia, a small group of islands called the Los Islands, and a strip of territory on the Nigerian frontier. The second document is called a declaration, and deals with Egypt and Morocco. In Egypt England gets from France freedom of action, the release of the surplus savings in Egypt hitherto held back, and simplification of finances; while France receives in return the neutralisation of the Suez Canal, 30 years' freedom of trade, and the continuation of French schools. In Morocco England gets 30 years' freedom of trade, and it is to her advantage that the coast between Melilla and the river Sebu is not to be fortified; France, on the other hand, is to have the right to preserve order and to make such arrangements with Spain as may be considered necessary to Spanish interests. The third document is called a *déclaration annexe*, and relates to Siam, Madagascar, and the New Hebrides. England consents to so interpret the Treaty of Siam that both parties will have complete liberty of action in their special zones outside the central valley of the Menam; she also agrees to the appointment of a Mixed Commission to decide land disputes between the natives in the New Hebrides; and in Madagascar the protest against a French Customs *régime* has been withdrawn.

### Death of Queen Isabella of Spain.

ON Saturday morning last ex-Queen Isabella of Spain passed away in Paris. The daughters of the ex-Queen, the Infantas Eulalia, Isabella, and Maria de la Paz, and her son-in-law, Prince Ludwig of Bavaria, were present at her death. During the morning M. Delcassé, Minister of Foreign Affairs, called to present his condolence and that of the French Government with the members of the late Queen's family. King Edward has since sent a message of sympathy. Marie Isabella Louisa, daughter of Ferdinand VII. of Spain, was born at Madrid on October 30th, 1820, and in 1821 was proclaimed Queen under the regency of her mother, Marie Christina, her father having issued before his death a Pragmatic decree revoking the Salic Law. The event led to the war of secession, which lasted seven years. The Cortes eventually confirmed the claims of Isabella by pronouncing sentence of exile on Don Carlos and his adherents. Queen Isabella was declared to have attained her majority in 1842, and was married to the elder son of her maternal uncle, Don Francisco d'Assisi, in 1846. In September 1868 a great revolution broke out in Spain, and the Queen retired to Pau, afterwards taking up her residence in Paris. On June 15th, 1875, she abdicated in favour of her son, Alphonse XII. Don Francisco, her husband and cousin, died a few years ago.

### The War in the Far East.

THE report that the Russians have entirely withdrawn from Northern Korea across the Yalu and Tumen rivers is officially confirmed. But it is asserted that they have laid mines along the north bank of the Yalu and along the coast as far as Ta-ku-shan. On Saturday the Marquis Ito made a speech at Tokio, in which he announced the complete attainment of the object of his mission to Korea, namely the establishment of relations of sincere trust and confidence between the two Courts. The Russian Minister at Pekin has again protested against the presence of Chinese troops on the Manchurian frontier, and demanded their withdrawal within the five-mile limit of the Great Wall. The city of Mukden is just now a sea of mud, and Admiral Alexeieff has been forced to take up his quarters in a private railway car.

### Painters in Water Colours.

THE Royal Society of Painters in Water Colours celebrates its centenary this year, and apparently in honour of the event the members and associates have contributed some of their best work. Altogether the show is a specially interesting one, and should not be missed. The President, Sir E. A. Waterlow, has some beautiful examples of peaceful English scenery, and it is difficult to decide which to admire most—"Summer Evening, Hampshire," "A Country Road," or "Mill, Hemingford Grey." Mr. John Sargent, R.A., the newest associate, has gone to Italy for his inspiration, "The Grand Canal, Venice." Mr. Melville has also been to the same source, and depicts "The Music Boat" as it passes under the Rialto Bridge. Another artist, Mr. R. W. Allan, paints the "Piazza del Popolo." Mr. Allan, however, is equally happy and realistic when he carries us up to Scotland, and portrays the fishing industry at "Portroy Harbour." The lady artists are well to the fore. Mrs. E. Stanhope Forbes contributes "April Evening"; Miss Fortescue Brickdale depicts a scene in a garden peopled with women of all ages, which she calls "Scandal"; Miss Rose Barton paints a corner of "Staple Inn, Holborn"; and Mrs. Allingham a country nook, "The Garden, Brocket, Hants."

### Spring Fashions.

WHEN Easter has come and gone fresh fashions are soon firmly established, and it is safe to indulge in new garments with a feeling of security. Coats and skirts are as much worn as ever, and the skirts are now "fashionably short" instead of "fashionably long." Short coatees are still worn, many so small that the sleeves only reach to the elbow, and though stoles are still in evidence, many coats are trimmed with quaint pointed capes. Coachmen's capes, split open and then joined together again with straps or buttons, are also much seen. When summer really comes we are told that we shall wear nothing but blouses of book muslin; but the weather, rather than fashion, will decide this point. Hats grow, if possible, flatter and flatter as to trimming, and the more a rosette has the appearance of being badly "sat upon" the better. The hats of one kind of straw, trimmed entirely—rosettes and all—with another coloured straw, are fashionable, useful, and becoming. Veils are voluminous, with a deep border or embroidered edge. These are gathered on a ribbon or a thread at the top, pulled up full, and then allowed to hang in festoons all round the hat.

### Notes and Queries.

**FOR REGULATIONS SEE PAGE 12.**

*Sanatoria.*

(14) Will you kindly give me the names, and fees if possible, of sanatoria in England where patients receive the open-air treatment.—*Amy.*

You will find a list in Burdett's "Hospital and Charities," and a list is also published by the National Association for the Prevention of Tuberculosis, Hanover Square.

*Home.*

(15) Can you tell me of a home, either free or for a small payment, where a girl aged ten of weak intellect could be admitted?—*Nurse B.*

Ascertain if she is eligible for the Royal Albert Asylum, Lancaster, by writing to the Secretary; if not, write to the Secretary of the National Association for Promoting the Welfare of the Feeble-minded, 53, Victoria Street, Westminster, S.W.

*Work. Retaining Fee.*

(16) (1) Where could a blind masseuse, trained by Mrs. Creighton Hale, apply for work? (2) Is it usual for country practitioners to charge a retaining fee of 10s. for a confinement, and two guineas if they are called in to attend the case?—*Seithy Tove.*

(1) The British and Foreign Blind Association, whose object is to promote the profitable employment of the blind, might help. Write to the Hon. Secretary, 33, Cambridge Square, Hyde Park, W. (2) A medical man has a right to make his own scale of charges.

*Mentone.*

(17) Will you give me the address of the Nursing Home at Mentone, and also any particulars as to the beginning of the season, &c.?—*E.E.S.*

English Nursing Institute, Avenue de la Gare, Mentone. The season lasts from the beginning of October to the end of April.

*Maternity Training.*

(18) Will the Editor kindly give names of maternity training schools, fees, &c. Is there a school which accepts ladies over 40?—*Sister S.*

Candidates for maternity training are accepted up to 45 years of age at the City of London Lying-in Hospital; up to 50 years of age at the Maternity Charity and District Nurses' Home, Howard's Road, Plaistow. For further information see "The Nursing Profession: Where and How to train," published by the Scientific Press.

*Massage.*

(19) Is there any place in London where the blind can be trained in massage?—*Nurse N.*

Write to the Secretary, the Incorporated Society of Trained Masseuses, 12, Buckingham Street, Strand, W.C.

*Charity for the Blind.*

(20 Will you kindly tell me if there is any fund from which a blind woman, 64, utterly destitute, would be eligible for a small weekly pension?—*Needful.*

Write full details to the Secretaries, The Blind Man's Friend Charity, 20, Old Burlington Street, W.; the Clothworkers' Company, 41, Mincing Lane, E.C.; Gardner's Trust for the Blind, 53, Victoria Street, S.W.; the Royal Blind Pension Society, 237, Southwark Bridge Road, S.E.; and to the Solicitors of Miss Harley's Charity for the Poor Blind, 19, Bedford Road, W.C.

**Important Nursing Textbooks.**

"The Nursing Profession: How and where to Train." 2s. net; 2s. 4d. post free.

"A Handbook for Nurses." By Dr. J. K. Watson. 5s. net; 5s. 4d. post free.

"Practical Guide to Surgical Bandaging and Dressings." By Wm. Johnson Smith, F.R.C.S. 2s. post free.

"The Nurses' Dictionary of Medical Terms and Nursing Treatment." By Honnor Morten. 2s. post free.

"Mental Nursing." By William Harding, M.D.Ed. 1s. post free.

"Art of Feeding the Invalid." (Popular Edition.) 1s. 6d. post free.

"On Preparation for Operation in Private Houses. By Stanhope Bishop, F.R.C.S. 6d. post free

"Modern Nursing in Private Practice." By Sir William Bennett. Price 7d. post free.

### For Reading to the Sick.

"HE WAS WOUNDED FOR OUR TRANSGRESSIONS."

When the cup of earthly gladness
Bears no taste of the life-giving stream
And high hopes, as though to mock our sadness,
Fade and die as in some fitful dream.

Who shall hush the weary spirit's chiding?
Who the aching void shall fill?
Who shall whisper of a peace abiding
And each surging billow calmly still?

Only He whose wounded heart was broken
With the bitter cross and thorny crown,
Whose dear love glad words of joy had spoken
Who His life for us laid meekly down.

Blessed Healer! all our burdens lighten;
Give us peace, Thine own sweet peace we pray;
Keep us near Thee till the morn shall brighten,
And all mists and shadows flee away.

*R. H. Baynes.*

However ill the world may go or seem to go, the Cross is the everlasting token that God so loved the world that He spared not His only begotten Son, but freely gave Him for it. Whatsoever else is doubtful, this at least is sure—that God must conquer, because God is God; that evil must perish, because God hates evil, even to the death.

*Charles Kingsley.*

We are all of us on the way of sorrows: life is a long up-hill, and "we go forth bearing our own cross." Each one of us has his own cross. Perchance we fall by the way: if so, we must arise, like our Lord, ready to try again, and so persevere to the mount of God and be faithful until death. O my God, give me this crowning grace of perseverance!—And on the way at least one Cyrenian shall be near to aid us in bearing our cross. Our Lord was glad of the help given to Him, and He aids us in turn, and bids us help one another, that not one of His little flock may lay aside his cross, but that all may cling to them with love until they are transformed into crowns.—*Anon.*

The fatigue of the spiritual life is a reality. Let go, then, the strain on thy inner being, and in the hour of spiritual fatigue go to the cross and gaze on the crucified form. Sit down and rest. It is such utter rest to lie at the sacred feet and gaze on Christ as our crucified Lord, for there clouds are scattered and strength is gained. In the wilderness of temptation we cling to the thought, "I am with Jesus here, for I am in Him,"—with the tenacious grasp of faith, in the hour when his presence is most unfelt.—*Anon.*

O, Son of God, Who camest from above
To take my flesh to bear my bitter cross;
Show me Thy tears, Thy tears of tender love
That I for Thee may count all gain but loss.

That I may know Thee, and by Thee be known;
That I may love Thee, and may taste Thy love,
That I may win Thee, and in Thee a crown;
That I may rest and reign with Thee above.

*H. Bonar, D.D.*

